# Non-fasting OGTT versus Fasting OGTT for screening of Hyperglycaemia in Pregnancy (HIP)

**DOI:** 10.12669/pjms.37.4.3979

**Published:** 2021

**Authors:** Shabeen Naz Masood, Nusrat Lakho, Saira Saeed, Yasir Masood

**Affiliations:** 1 Dr. Shabeen Naz Masood, MBBS, MCPS, FCPS, PhD, DCPS. Department of Obstetrics & Gynaecology, Isra University, Karachi, Pakistan; 2 Dr. Nusrat Lakho, MBBS, FCPS. Department of Obstetrics & Gynaecology, Isra University, Karachi, Pakistan; 3 Dr. Saira Saeed, MBBS, FCPS. Department of Obstetrics & Gynaecology, Isra University, Karachi, Pakistan; 4 Dr. Yasir Masood, MD, MPH. Department of Public Health, Brown School, Washington University in St. Louis, Missouri, USA

**Keywords:** Hyperglycemia in Pregnancy (HIP), Fasting OGTT, Non Fasting OGTT, GDM, Diabetes in Pregnancy (DIP)

## Abstract

**Objectives:**

To compare the reliability of non-fasting oral glucose tolerance test (OGTT) versus fasting OGTT for screening of hyperglycaemia in pregnancy (HIP).

**Methods:**

This cross sectional analytic study was conducted by the Department of Obstetrics & Gynaecology, Isra University Karachi Campus from October 2016 to April 2017. A total of 225 pregnant women irrespective of gestational age were included in the study. They underwent non fasting 75 grams OGTT. Venous plasma glucose was done 02 hours after the glucose load. Same women were advised to come again within three to seven days for fasting OGTT. Venous plasma glucose (VPG) was estimated in fasting and 2 hours post glucose load.

**Results:**

Using the non-fasting OGTT, out of 204 women, 32 were diagnosed with hyperglycemia. All these 204 women were again called for fasting OGTT three to seven days after the initial non-fasting OGTT. Only nine were diagnosed with hyperglycemia, out of these nine women, seven women who were screen positive on non-fasting OGTT were found to be screen positive on fasting OGTT as well. However, only two women were additionally diagnosed with hyperglycemia who were initially screen negative on non-fasting OGTT. The non-fasting OGTT has diagnosed HIP with sensitivity of 77.7%, specificity of 87.1%, positive predictive value of 21.8% and negative predictive value of 98.8%.

**Conclusion:**

The use of the non-fasting OGTT at first antenatal visit may be a practical approach to detect the HIP as screening and diagnostic tool in the resource constrained settings.

## INTRODUCTION

The prevalence of diabetes is increasing with almost equal proportions in men and women. Currently it is reported as high as 26.3% in developing countries like Pakistan.^1^ Gestational Diabetes Mellitus (GDM) is the most common medical disorder of pregnancy. It is reported to affect 15% of pregnant women worldwide.^2^-^4^

Maternal adiposity, insulin desensitizing effects of placental hormones,^5^,^6^ genetic and epigenetic factors predispose Asian women to develop diabetes in pregnancy.^7^ In the absence of specific symptoms of diabetes in pregnancy, there is a need for universal screening of blood glucose in pregnancy. The screening method should be sensitive, reliable and patient friendly. It should also be diagnostic, cost effective and affordable in resource constraint settings.

The fasting OGTT recommended by World Health Organization (WHO) and IADPSG is considered as standard and confirmatory test to diagnose HIP.^8^ However, it requires women to come in fasting state,^9^ or stay fasted while waiting to be seen in busy clinical and laboratory settings. Multiple blood test and misconceptions about prolonged fasting and long travel distance to health facilities leads to non-compliance and inconvenience.Non-fasting OGTT is a modified version of WHO criteria, is one-step procedure, does not require fasting and requires single blood sample for blood glucose screening.

The rationale of performing non fasting OGTT is that, normoglycaemic women usually have normal insulin response when given glucose load, whereas a pregnant woman with diabetes will not be able to maintain euglycemia post meals and after glucose load. This may be due to higher insulin resistance and impaired insulin secretion.^10^

 Non-fasting OGTT is more acceptable to women as they do not have to drink glucose solution empty stomach, causes less nausea and vomiting in pregnancy and involves less operational cost. IADPSG criteria requires multiple blood samples, extra cost, men power and additional resources which is an added burden to developing countries working with marginal health economies. However, the universal BG screening recommended by IADSPG panel of experts is less likely to miss any women with GDM and is a recognized screening methodology where resources permit.^11^

### Rationale of study

In the present study non-fasting OGTT was compared with fasting oral glucose tolerance test to evaluate the sensitivity and specificity of non-fasting OGTT versus fasting OGTT.

## METHODS

This cross-sectional study was done to evaluate sensitivity and specificity of non-fasting blood glucose screening versus fasting OGTT as the gold standard. This study was done as a part of World Diabetes Foundation (WDF) GDM Project in Pakistan (WDF 14-896). It was conducted at outpatient Department of Obstetrics and Gynaecology, Isra University Karachi Campus from October 2016 to April 2017.

### Ethical approval

This study was approved by the Institutional Ethics Review Committee of Al-Ibrahim Eye Hospital, Isra University, Karachi Campus, Pakistan, with protocol number A-00076/2 on 3^rd^ March 2017.

Sample size was calculated from software openepi.com by taking statistical conditions of 5% margin of error and 95% confidence interval. Hypothesized % frequency of outcome factor in the population prevalence P=26.3% was cited from the study Reference #2 IDF Diabetes Atlas. 6^th^ ed. The required sample size was derived to be 225. The formula used in sample size calculation was mentioned below:

*Sample size:*

n = [DEFF*Np(1-p)]/ [(d^2^/Z^2^_1-α/2_*(N-1)+p*(1-p)]

### Sample Size

According to study design, following formula was used to calculate the sample size.


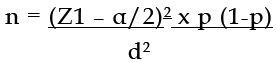


The required sample size was derived to be 225 women.

### Inclusion Criteria

All pregnant women who consented for the study were included at first antenatal visit irrespective of gestational age and associated comorbidities for blood glucose screening.

### Exclusion Criteria

Pregnant women with pre-existing / diagnosed / confirmed cases of diabetes.

The data was collected on prespecified questionnaire by trained medical officer. Women were given 75 grams of glucose in 250 cc of water irrespective of last meal to drink it slowly over a period of 3-5 minutes. Zero hour was considered when the women first sipped the glucose solution. Venous blood sample was collected after two hours of oral glucose load. The samples were centrifuged immediately and analyzed by the glucose peroxidase method using commercially available kit provided by Randox. All women who were subjected to non-fasting OGTT were requested to come back within 3-7 days with eight hours fasting. Venous blood sample was taken in fasting state and another blood sample was drawn two hours after 75 gm glucose load. The cut off for fasting blood sugar and two hours post glucose load was taken as >126 and >140 mg/dl respectively (WHO criteria).

### Statistical Analysis

Statistical analysis was performed using Statistical Package for Social Sciences (SPSS) software for windows version 20.0. All continuous variables were presented as mean± standard deviation. Categorical variables were presented as frequency and percentages. Diagnostic accuracy of non-fasting OGTT and fasting OGTT was shown in 2x2 contingency table with sensitivity, specificity, positive predictive values and negative predictive values. To know the significance between different variables Chi-square test was applied. P-value ≤ 0.05 was considered to be statistically significant.

## RESULTS

Two hundred and twenty-Five, pregnant women were subjected to non-fasting OGTT, 21 women were dropped from the study because of vomiting and non-compliance. Using the non-fasting OGTT, out of 204 women, 32 were diagnosed with hyperglycemia. All these 204 women were again called for fasting OGTT 3—7 days after the initial non-fasting OGTT. Only nine were diagnosed with hyperglycemia, out of these nine women, seven women who were screen positive on non-fasting OGTT were found to be screen positive on fasting OGTT as well. However, only two women were additionally diagnosed with hyperglycemia who were initially screen negative on non-fasting OGTT. Fasting hyperglycaemia was detected in only one (0.7%) woman with fasting OGTT.

Most of study participants were below 31 years of age with mean age of 25.5 with standard deviation of 9.3. Among the screen positive women, 7 (21.9%) were less than 25 year of age, 13 (40.6%) were 26-30 years and 12 (37.5%) were more than 31 years of age. The mean age of screen positive women was 29 years and that of screen negative women was 26.9 years.

Women attended antenatal clinics at different periods of gestation, out of 32 screen positive women, 11 (5.3%) attended outpatient department before 14 weeks of gestation, 18 (8.8%) between 15 to 28 weeks and 3 (1.4%) after 28 weeks of gestation. Mean gestational age at first antenatal visit was 21 weeks.

**Table-I:** Comparison of Diagnostic accuracy of non-fasting versus fasting OGTT (n = 204)

		Fasting OGTT		Total

		GDM +ve	GDM –ve	
Non-fasting OGTT	Test +ve	7	25	32
	Test -ve	2	170	172
Total		9	195	204

Sensitivity 77.78%, Specificity 87.18%, PPV 21.88%, NPV 98.84%.

The non-fasting OGTT method has diagnosed HIP with sensitivity of 77.7%, specificity of 87.1%, positive predictive of 21.8% and negative predictive value of 98.8%.

## DISCUSSION

The prevalence of HIP is reported variably from 1.4 to 14% worldwide among different racial and ethnic groups. Prevalence is higher in Asian women than in Caucasian women.^12^ In Pakistan, prevalence of HIP has been reported from 4.2% to 26% due to wide variations in screening and diagnostic criteria.^13^,^14^ The prevalence of HIP in present study was 4.4% by fasting OGTT. While in South and North India, prevalence of HIP was between 6 and 7%.^15^,^16^ Similar results were found in a study with the prevalence of 7.6% in US women.^17^

Increasing maternal age is an established risk factor for GDM. There was statistically significant difference in age between hyperglycemic and normoglycemic women. In present study the mean age of screen positive women was 29 years. Similarly, in a study by Sharma A et al.^18^ GDM was observed in 27.4% of women in age group of >30 years. The study by Terence T Lao et al.^19^ observed that risk of GDM increases significantly and progressively higher from 25 years onwards.

**Fig.1:**
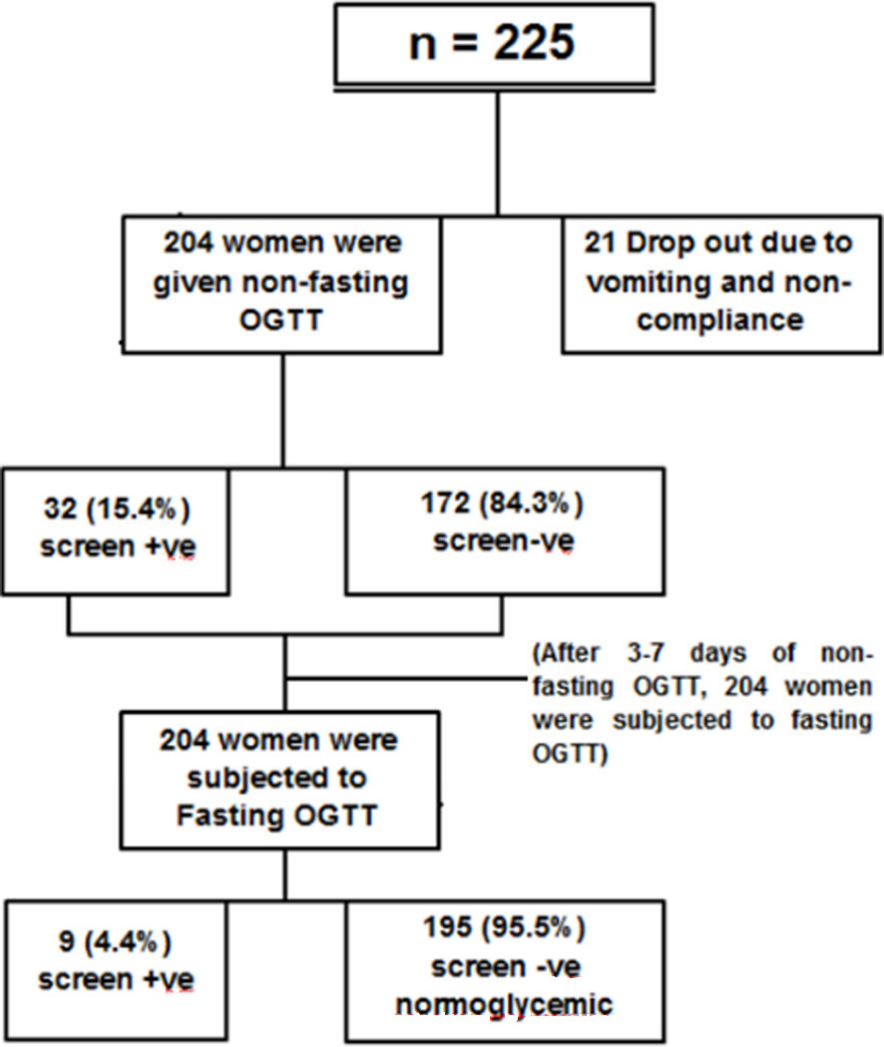
Consort Diagram.

**Table-II:** Demography of study participants subjected to non-fasting OGTT (n = 204).

Parameters		Non-fasting OGTT		*P*-value

		+ve (n=32)	-ve (n=172)	
Age years	<25 Years	7	28	0.718
		21.9%	16.3%	
	25-30 years	13	79	
		40.6%	45.9%	
	>30 years	12	65	
		37.5%	37.8%	
Parity	>=4	12	47	0.244
		37.5%	27.3%	
	< 4	20	125	
		62.5%	72.7%	
Family history of diabetes	Father	6	15	0.000
		18.8%	8.7%	
	Mother	7	66	
		21.9%	38.4%	
	Siblings	0	39	
		0.0%	22.7%	
	No Family History	19	52	
		59.4%	30.2%	
Previous past history of gestational diabetes mellitus	Yes	1	5	0.05
		3.1%	2.9%	
	No	29	166	
		90.6%	96.5%	
	Don’t Know	2	1	
		6.3%	0.6%	
Gestational Age weeks	<14 weeks	11	37	0.037
		34.4%	21.5%	
	14-28 weeks	18	83	
		56.3%	48.3%	
	>28 weeks	3	52	
		9.4%	30.2%	

Chi-square test was applied with *p*-value 0.5 was considered as significant.

The risk of GDM increases with increasing gestational age because of increased placental contra-insulin hormones and increasing insulin resistance, it is because of this that the usual recommendations for GDM screening is between 24 and 28 weeks of gestation.^20^

According to the latest National Survey,^1^ almost 25% of the population in Pakistan is suffering from Type-2 diabetes mellitus (T2DM), 76.2% and 62.1% are overweight and obese, respectively. Obesity, higher body mass index (BMI), and insulin resistance are added risk factors for undiagnosed diabetes in early pregnancy.^21^ Metabolic testing outside the pregnancy is not commonly performed, this increases the need for early screening during pregnancy to identify and treat dysglycemia before the period of rapid fetal organogenesis from 0 to 8 weeks to avert congenital fetal anomalies.^22^

In present study 34.4% of women were detected with hyperglycaemia who attended antenatal clinic in first trimester. It is necessary to initiate screening earlier in pregnancy as undiagnosed and untreated hyperglycemia during organogenesis can be toxic to fetal DNA and may increase the risk of fetal congenital anomalies.^9^

In this study majority of women attended antenatal clinic late in second trimester of gestation and 53.6% of them were found to be screen positive. The detection rate similarly increased by 44.4%,^23^ 38%,^24^ and 31.8%,^13^ with increasing period of gestation in other studies as well.

In present study there is inverse relationship of parity and GDM, 62.5% of screen positive women were having less than four children whereas studies show increased risk of developing GDM with increasing age and parity.^25^-^27^

Family history of diabetes is a major risk factor for development of GDM.^10^,^18^,^28^ In this study, family history of diabetes was present in about 13 (40.6%) women, whereas 20% of their parents were found to have diabetes, while their siblings did not give any history of diabetes. In present study 90.6% of screen positive women did not give previous history of GDM, possible reasons could be that women did not know or were not screened in previous pregnancies or due to lack of uniform screening methodology. Other studies also report that women did not know about previous history of GDM.^1^, ^29^

In present study the sensitivity of non-fasting OGTT compared with fasting OGTT was 77.7% and specificity 87.1%. Similar results were observed with sensitivity of 76.1% and specificity of 96.3% in study conducted by Tripathi R et al.^30^ Using non-fasting OGTT other studies also reported 98-100% sensitivity and specificity with no statistically significant difference (P>0.05) between non fasting and fasting OGTT.^10^,^15^,^31^ The higher sensitivity seen in these studies may be because of risk based selection and women were screened in advanced gestational age. A recent study by Mohan V et al,^32^ has shown low sensitivity of non-fasting OGTT compared to the fasting OGTT (sensitivity 22.6%, specificity 97.8%).

The important finding in this study was that out of 204 study subjects only one woman had fasting hyperglycemia. Other studies also show that the fasting plasma glucose is inherently much lower in Asians than Caucasians as compared to post prandial values.^33^,^34^ In present study there were substantial number of women (10.7%) who were detected as false positive by non-fasting OGTT, this may cause unnecessary apprehension and anxiety in the women and the family as the pregnancy is not a good time to give bad news. In spite of this low positive predictive value (PPV 21.8%) of single non-fasting OGTT, this can be considered as a pragmatic screening test in view of its high negative predictive value (98.84%) with less chances of missing true cases.

### Limitations of the study

It is a cross sectional single centered study with a limited generalizability. Further multicenter studies may be conducted with larger sample size.

### Strength of the study

The strength of the study was that all study participants were subjected to fasting and non-fasting blood glucose test, which reduced the bias and may have improved sensitivity and specificity of the screening test.

## CONCLUSION

The use of the non-fasting OGTT as a universal single step screening test at first antenatal visit may be a practical and pragmatic approach to detect HIP in the resource constrained settings as screening and diagnostic tool.

High sensitivity is important for any screening test but higher negative predictive value also has an important role so that true cases are not missed. This study supports the concept of universal screening using single non-fasting OGTT for screening and diagnosis of HIP with less chances of missing GDM. Where resources permit, fasting OGTT or IADPSG criteria may be undertaken as per global recommendations and practices.

### Authors’ Contribution:

**SNM & NL:** Conception, design, drafting and intellectual content analysis.

**SS:** Conception and acquisition of data.

**YM:** Manuscript design, interpretation of data and statistical analysis.

**SNM:** Responsible and accountable for the integrity of the work..
